# Diagnostic Performance of Echocardiography for the Detection of Acute Cardiac Allograft Rejection: A Systematic Review and Meta-Analysis

**DOI:** 10.1371/journal.pone.0121228

**Published:** 2015-03-30

**Authors:** Wei Lu, Jun Zheng, Xudong Pan, Lizhong Sun

**Affiliations:** 1 Department of Cardiac surgery, Beijing Anzhen Hospital, Capital Medical University, Beijing, 100029, China; 2 Beijing Institute of Heart, Lung and Blood Vessel Diseases, Beijing, 100029, China; Centre for Inflammation Research, UNITED KINGDOM

## Abstract

**Objective:**

Many studies have addressed the diagnostic performance of echocardiography to evaluate acute cardiac allograft rejection compared with endomyocardial biopsy. But the existence of heterogeneity limited its clinical application. Thus, we conducted a comprehensive, systematic literature review and meta-analysis for the purpose.

**Methods:**

Studies prior to September 1, 2014 identified by Medline/PubMed, EMBASE and Cochrance were examined by two independent reviews. We conducted meta-analysis by using Meta-DiSc 1.4 software. An assessment tool of QUADAS-2 was applied to evaluate the risk of bias and applicability of the studies.

**Results:**

Thirty studies met the inclusion criteria of meta-analysis. The four parameters of pressure half time, isovolumic relaxation time, index of myocardial performance and late diastolic mitral annular motion velocity were included in the meta-analysis, with a pooled diagnostic odds ratio of 10.43, 6.89, 15.95 and 5.68 respectively, and the area under the summary receiver operating characteristic curves value of 0.829, 0.599, 0.871 and 0.685 respectively.

**Conclusion:**

The meta-analysis and systematic review demonstrate that no single parameter of echocardiography showed a reliable diagnostic performance for acute cardiac allograft rejection. A result of echocardiography for ACAR should be comprehensively considered by physicians in the context of clinical presentations and imaging feature.

## Introduction

Presently, heart transplantation (HTX) is the only effective treatment modality for end-stage heart diseases. Despite the improvements of prognosis for HTX patients over the past 20 years, acute cardiac allograft rejection (ACAR) remains the most common complication during the first year after transplantation. Approximately 40% of patients will experience at least one episode of ACAR within this period. Furthermore, ACAR contributes to approximately 12% of mortality between 1 and 12 months of post-transplantation, and was an independent risk factor for developing into cardiac allograft vasculopathy (CAV), an irreversible stage to final allograft dysfunction. Even with effective treatments, an episode of ACAR occurring in the first year will increase two-year and four-year fatalities [1]. Therefore, early detecting and curbing ACAR is essential to the survival of HTX patients.

However, clinical features of ACAR are not consistent, for patients usually remain asymptomatic until hemodynamic compromise occurs. Invasive surveillance procedure is obligatory to perform routinely and frequently in order to detect ACAR in an earlier stage. Timely diagnosis following by early immunosuppressive treatment, will prevent rejection from developing into more severe grade, with the aim of achieving better long-term result [1]. Right ventricular endomyocardial biopsy (EMB) still represents the clinical gold standard in monitoring cardiac allograft rejection. Nevertheless, this invasive diagnostic procedure is uncomfortable and concomitant with several, albeit rare, major complications such as carotid artery puncture, cardiac tamponade and permanent heart block. EMB also has a number of limitations like sample error and myocardial scarring[2,3]. Non-invasive but equally accurate technique to detect rejection is highly desirable.

Many promising modalities have been tried to develop a sensitive and specific non-invasive method. Of the many diagnostic techniques, echocardiography is the most ubiquitous tool for monitoring ACAR since it is easily performable, time-saving, and not associated with the risks of the invasive methods [4]. Its versatility allows it to be applied in a wide variety of circumstances during the post-transplant period. Some studies have addressed the diagnostic accuracy of echocardiography to assess the rejection grade of ACAR compared with EMB. But the methodological heterogeneity, such as different parameters and cutoff value, which led to conflicting outcomes among individual studies, limited the clinical application of echocardiography. It is necessary to further assess the diagnostic value of echocardiography for the detection of ACAR. Accordingly, we seek a comprehensive, systematic literature review and meta-analysis for the purpose.

## Methods

### Study Protocol

The analysis complied with a predetermined protocol [5]. The data were collected according to Preferred Reporting Items for Systematic Reviews and Meta-Analysis (PRISMA) (S1 PRISMA Checklist).

### Data resource and Search strategy

We systematically searched the Cochrance clinical trials database, Medline/Pubmed and EMBASE to identify eligible studies prior to September 1, 2014. No starting date was limited. In addition to database searches, we reviewed the references of included studies and other relevant review articles to obtain a comprehensive list of included studies. Two authors (W.L. and J.Z.) searched and reviewed database independently. Disagreements were resolved by discussion or upon consensus from a third reviewer. We used the following Medical Subject Headings and search terms: “echocardiography,” “heart transplantation” and “graft rejection.” The searching formula is shown below

#### Medline search formula

((("Echocardiography"[Mesh]) OR (echocardiogra* OR sonogra* OR ultrasonic* OR doppler OR echo OR ultrasound OR ultrasonogra*))) AND ((((("heart transplantation"[MeSH Terms] OR heart transplantation[Text Word])) AND ("graft rejection"[MeSH Terms] OR graft rejection[Text Word]))) OR ((cardiac OR heart) AND (transplantation OR transplant OR allograft* OR graft*) AND (rejection* OR reject)))

#### Embase search formula

'Echocardiography' OR 'echocardiography'/exp OR echocardiography OR echo OR ultrasonic OR doppler OR ultrasound AND ('heart' OR 'heart'/exp OR heart OR cardiac) AND ('transplantation' OR 'transplantation'/exp OR transplantation OR transplanted OR transplant OR 'allograft' OR 'allograft'/exp OR allograft) AND (rejection OR reject) AND ([article]/lim OR [article in press]/lim OR [review]/lim) AND ([article]/lim OR [article in press]/lim) AND [english]/lim AND [humans]/lim AND [embase]/lim.

### Study selection

Selection criteria ① for Meta-analysis: (I) Type of study: Diagnostic accuracy test. (II) Population: Underwent HTX with all age spectrums. (III) Index test: echocardiography. (IV) Reference standard: EMB. (V) Language: Published in English. (VI) True-positive (TP), false-positive (FP), true-negative (TN) and false negative (FN) data were available or could be derived from articles.

Selection criteria ② for summarization: (I) Type of study: Diagnostic accuracy test. (II) Population: Underwent HTX with all age spectrums. (III) Index test: echocardiography. (IV) Reference standard: EMB. (V) Language: Published in English.

Exclusion criteria: (I) Type of study: Reviews, case reports, editorial, conference presentations or animal researches. (II) Sample size <10 patients. (III) Duplicated data.

### Data extraction and quality assessment

The following variables were extracted from each study: author, publication year, country, demographic characteristics of study population, study design (prospective or retrospective), recruitment method (consecutive or random), interval between echocardiography and EMB, blind, echocardiographic parameter, cutoff value, rejection grade of detection, reference of histological interpretation for rejection grade, and number of TP, FP, TN and FN. If studies enrolled all of subjects during a certain period, and conducted echocardiogrphy and EMB on them, the recruitment method will be defined as “consecutive”, even if the studies did not describe the method. Two authors extracted the data from eligible studies independently (W.L. and J.Z.). The methodological quality of the eligible studies was assessed by two authors (XD.P. and J.Z.) independently using the Quality Assessment of Diagnostic Accuracy Studies-2 (QUADAS-2), an assessment tool used in systematic reviews to evaluate the risk of bias and applicability of primary diagnostic accuracy studies [6]. In the same way, disagreements were resolved by discussing together or appealing to a third author.

### Data synthesis and statistical analysis

Meta-DiSc version 1.4 [7] statistical software was used for our study. Analysis process included four steps as follows. First of all, Spearman correlation coefficient between sensitivity (se) and specificity (sp), and p-value, were computed to explore heterogeneity arising from threshold effect. Subgroup analysis would be conducted according to different threshold variables. Secondly, non-threshold heterogeneity was explored by using inconsistency (I2) value and χ2 test [8]. I2 value within 25–49%, 50–74% or 75–100% was considered a low, moderate or high degree of heterogeneity respectively [9]. Subsequently, sensitivity analysis was applied to explore the source in case of the existence of non-threshold heterogeneity, and DerSimonian-Laird random effects model was considered if necessary [10]. Otherwise, pooled sensitivity, specificity, positive likelihood ratio (PLR), negative likelihood ratio (NLR), diagnostic odds ratio (DOR), and area under the curve (AUC) with 95% confidence interval (CI) were calculated by using Mantel-Haenzsel fixed effects models [8]. The pooled DOR was used for constructing summary receiver operating characteristic curve (SROC), with its Q point representing the maximal joint of sensitivity and specificity [11,12]. We also summarized all of the articles which met the selection criteria ②. If a study showed a certain index correlated with ACAR, the index would be marked as “+”, otherwise, marked as “-”.

## Results

**Fig 1:**
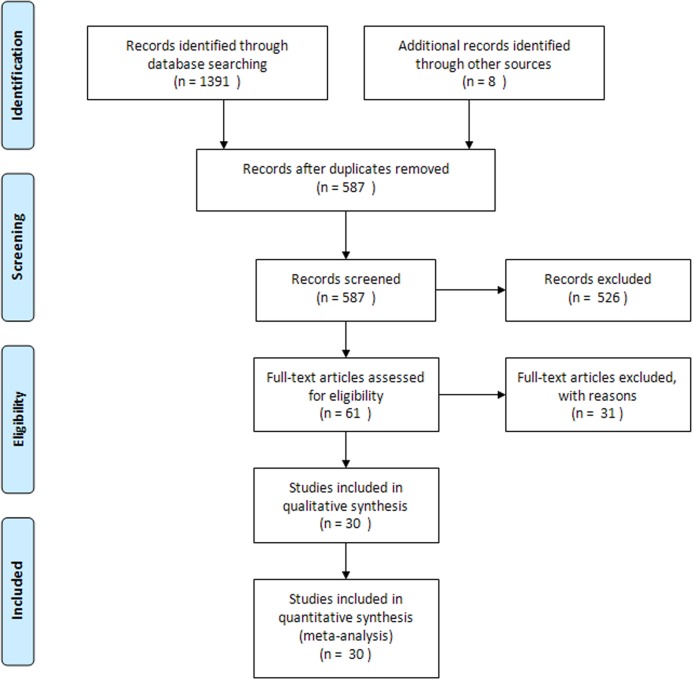
Flow diagram of study selection process.

Database search and additional citation tracking of review and original articles produced 1391 potentially relevant citations, 683 from Medline/Pubmed, 708 from EMBASE and 0 from Cochrane library. After getting rid of ineligible articles, such as duplicated articles, case reports, reviews or animal researches, we submitted 61 studies for a full text review. Thirty one studies were excluded, in which, 16 articles failed to construct 2×2 table, 4 did not focus on diagnostic test, and 11 were correlated with cardiac allograft vasculopathy. Finally, 30 eligible studies were included in meta-analysis. The selection process is presented in ***Fig. 1***.

### Characteristics and quality of included articles

Thirty articles for meta-analysis were published during a long span of time, from 1988 to 2014 [13–42]. Prospective studies account for 73.3% (22/30) of all eligible studies. A total of 2100 patients was included in the analysis. Characteristics of included studies are shown in ***Table 1***. Of all the studies, 15 studies (50.0%) consecutively recruited subjects for research. There are 11 studies (36.7%) complying with double-blind principle when interpreting index test and reference standards. Twenty seven studies (90.0%) performed EMB and echocardiography on the same day. EMB was used as a reference standard in all eligible studies. However, two studies included an additional clinical reference standard that patients presenting hemodynamic compromise, even with a negative histological result, were deemed to have ACAR [15,19]. Eight studies (26.7%) performed echocardiography using a pre-specified threshold value. Seven articles (23.3%) enrolled pediatric patients as research subjects. The risk of bias and applicability of the studies was evaluated based on QUADAS-2 summarized in S1 Fig. and S2 Fig.

**Table 1:** Characteristics of eligible studies for meta-analysis.

Study	Year	Country	Design	Study population	Enrollment method	Blind	Sample size	Interval	Dectecion of grade	Parameters	Cutoff value
Angermann [13]	1997	Germany	P	Adult	Consecutive	Single	52	0	≥IB	PWED 2D-IB	1.5dB^a^
									≥IIIA	PWED 2D-IB	5.5dB^a^
Asante-Korang [14]	2003	USA	P	Child	Consecutive	Unclear	20	0	≥IIIA	Em/Am	1
Behera [15]	2007	USA	R	Child	Unclear	Double	148	0	≥IB, AMR, clinical rejection	E/Em	5
Ciliberto [16]	1994	Italy	P	Adult	Unclear	Single	130	0	≥IA	PHT, IVRT	20ms
Ciliberto [17]	1996	Italy	P	Adult	Unclear	Single	30	0	≥IA	Multiple parameters	
Desruennes [18]	1988	France	P	Adult	Unclear	Unclear	26	0	≥IA	PHT, IVRT	20% decrease
Dandel [19]	2001	Germany	P	Adult	Unclear	Single	293	Unclear	≥II, clinical rejection	Sm,Em	10% decrease
Fauchier [20]	1997	France	P	Adult	Unclear	Double	23	0	≥IA	PHT	20% decrease
Flanagan [21]	2013	USA	R	Child	Unclear	Double	80	<3 days	≥II	Relative change of IMP	≥20.4%
Kato [22]	2010	Japan	P	Adult	Unclear	Single	35	0	≥IB	Systolic strain	−27.4%
Lieback [23]	1994	Germany	P	Adult	Unclear	Single	23	0	≥IB	Multiple parameters	
Leonard [24]	2005	USA	P	Child	Consecutive	Unclear	21	0	≥II	IMP	<0.44
Lunze [25]	2013	USA	R	Child	Consecutive	Unclear	122	0	≥II, AMR	Relative change of Sm	15%^b^
										Relative change of Am	5%^b^
Mankad [26]	1999	USA	P	Adult	Consecutive	Double	78	0	≥IB	Sm+Em	135mm/s
Moidl [27]	1998	Austria	R	Adult	Unclear	Single	94	0	≥II	PFR	<4 EDV/s
Mouly-Bandini [28]	1996	France	P	Adult	Unclear	Double	23	0	≥IA	PHT, IVRT	20% decrease
Marciniak [29]	2007	Belgium	P	Adult	Consecutive	Single	31	0	≥IB	Radial strain of LVPW	≤30%
Moran [30]	2000	USA	P	Child	Consecutive	Single	37	0	≥IIIA	Stress velocity index	−2
Palka [31]	2005	Australia	P	Adult	Consecutive	Double	44	0	≥IIIA	IVRMVG	>0.1S^−1^
Putzer [32]	2000	USA	P	Child	Consecutive	Unclear	18	0	≥IIIA	ECHO-B score	≥4
Park [33]	1992	Germany	P	Adult	Unclear	Single	96	0	≥II	Te	20% increase
Pan [34]	2011	China	P	Adult	Consecutive	Single	95	0	≥II	Tmsv 16-SD%	1.73
Roshanali [35]	2010	Iran	P	Adult	Unclear	Double	38	0	≥IIIA	(PWT + LVMI)-(Lat-S + Sep-TS)	1
Resende [36]	2011	Brazil	P	Adult	Consecutive	Double	54	0	≥IIIA	Am	7% decrease
Stengel [37]	2001	Swizerland	P	Adult	Consecutive	Double	41	0	≥IIIA	Am	<8.7cm/s
Sera [38]	2014	USA	R	Adult	Unclear	Unclear	59	0	≥IB	Global longitudinal strain	<14.8%
Sun [39]	2005	USA	P	Adult	Consecutive	Single	264	0	≥IB	Am	<9cm/s^b^
										IVRT	<90ms^b^
Sato [40]	2011	Japan	P	Adult	Consecutive	Single	32	0	≥II	LV systolic torsion	25% decrease
Toumanidis [41]	2003	Greece	R	Adult	Consecutive	Double	24	0	≥IA	IMP	<0.69
Vivekananthan [42]	2002	USA	R	Adult	Unclear	Double	40	0	≥IIIA	IMP	20% decrease

A, late diastolic mitral inflow velocity; Am, Late diastolic mitral annular motion velocity; AMR, antibody mediated rejection; Double, Echo blind to EMB and EMB blind to Echo; E, early diastolic mitral inflow velocity; Em, early diastolic mitral annular motion velocity; IMP, index of myocardial performance; Interval, interval between Echo and EMB; IVRMVG, Peak late isovolumic relaxation myocardial velocity gradient; IVRT, isovolumic relaxation time; LVPW, left ventricular posterior wall; LVMI, LV mass index; Lat-S, LV lateral peak systolic strain; P, Prospective; PWED 2D-IB, Posterior wall End-diastolic 2 dimension-Intergrated backscatter; PHT, pressure half time; PFR, left ventricular peak filling rate; PWT, LV posterior wall thickness; R, retrospective; Single, Echo blind to EMB or EMB blind to Echo; Sep-TS, septum time to systole; Sm, systolic mitral annular motion velocity; Te, time interval between maximal posterior wall contraction and the point of peak posterior wall endocardium retraction velocity; Tmsv 16-SD, standard deviation of time to minimum systolic volume of ventricular segment of 16 segments.

^a^, cutoff value corresponding to different rejection grade.

^b^, cutoff value corresponding to different parameters.

### Diagnostic information and accuracy

A total of 41 parameters [e.g. Pressure half time (PHT), isovolumic relaxation time (IVRT), index of myocardial performance (IMP), late diastolic mitral annular motion velocity (Am), early diastolic mitral annular motion velocity (Em)] was able to construct 2×2 table. Among them, the parameters of diastolic function accounted for 48.8% (20/41), and 29.2% (12/41) of parameters reflected systolic function. Seven studies supplied se and sp by combining two or more parameters to detect ACAR. Finally, the parameters of PHT, IVRT, IMP and Am were included in the meta-analysis, because the number of eligible studies using the other parameters is less than 3.

### PHT

**Fig 2:**
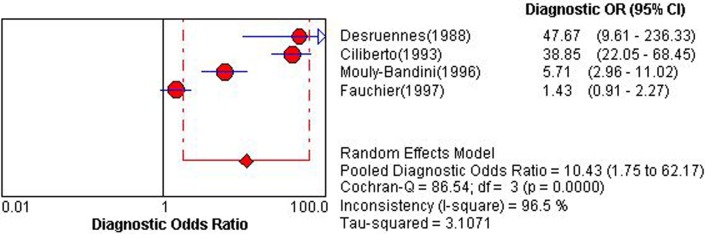
Pooled diagnostic odds ratio of pressure half time for detecting acute cardiac allograft rejection. Random-effects models were applied to pool effect sizes. Each solid diamond represents a value of pooled diagnostic odds ratio. Sample size is indicated by the size of the square. CI, confidence interval; df, degrees of; OR, odds ratio.

**Fig 3:**
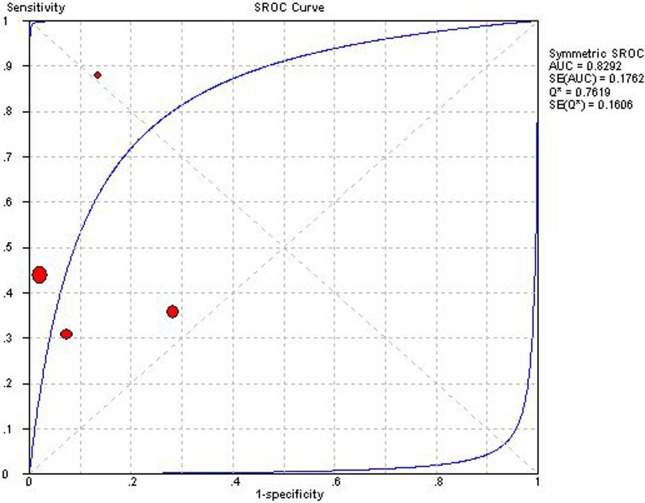
Summary receiver operator characteristic of pressure half time. The figure shows a symmetric curve with an area under the curve of 0.8292 and standard error of 0.1762. Each study is represented as a square in the summary receiver operating characteristic. The sample size is shown by the size of the square. AUC, area under the curve; SROC, Summary receiver operator characteristic; SE, standard error.

Four studies using PHT for detection of ACAR could construct 2×2 table [16,18,20,26]. Overall diagnostic performance of PHT was shown in ***Figs. 2 and 3***. The Spearman correlation coefficient was computed as a result of 0.00 with a *P* value 0.10, which suggested the absence of a threshold effect. For the existence of a high degree of heterogeneity, DerSimonian-Laird random effects model was introduced to pool diagnostic indices. Pooled se, sp, PLR, NLP, DOR and AUC of SROC curve was 0.41 (0.36–0.47), 0.91 (0.90–0.93), 5.26 (1.23–22.44), 0.65 (0.48–0.88), 10.43 (1.75–62.17) and 0.829, respectively. Sensitivity analysis demonstrated the high degree of heterogeneity was consistent after each study was removed, which indicated the heterogeneity might arise from multiple factors.

### IVRT

**Fig 4:**
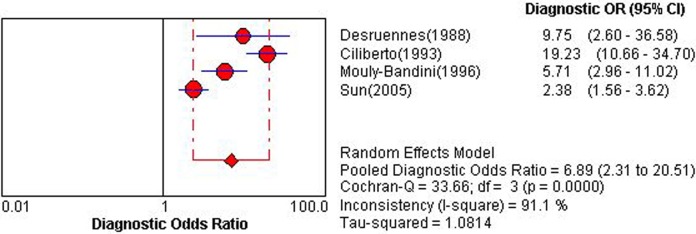
Pooled diagnostic odds ratio of isovolumic relaxation time for detecting acute cardiac allograft rejection. Effect sizes were pooled by random-effects models.

**Fig 5:**
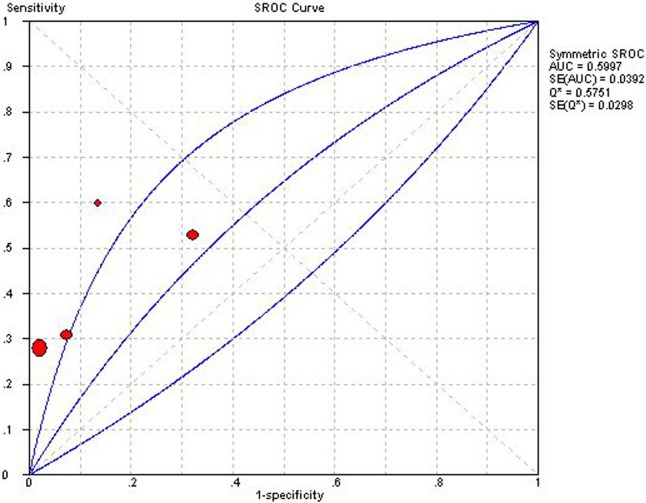
Summary receiver operator characteristic of isovolumic relaxation time. The figure shows a symmetric curve with an area under the curve of 0.5997 and standard error of 0.0392.

Four studies employing IVRT could construct 2×2 table [16,18,26,38]. Overall diagnostic performance of IVRT was shown in ***Figs. 4 and 5***. The Spearman correlation coefficient was computed as a result of 0.80 with a *P* value 0.20, which suggested no existence of a threshold effect. Except for the absence of heterogeneity in NLR, the other indices showed a high degree of heterogeneity. Therefore, DerSimonian-Laird random effects model was used for pooling diagnostic indices. Pooled se, sp, PLR, NLP, DOR and AUC of SROC curve was 0.40 (0.35–0.45), 0.92 (0.90–0.93), 4.54 (1.51–13.62), 0.72 (0.66–0.79), 6.89 (2.31–20.51) and 0.599, respectively. Sensitivity analysis demonstrated the high degree of heterogeneity was consistent after each study was ruled out, which showed the heterogeneity might arise from multiple factors.

### IMP

**Fig 6:**
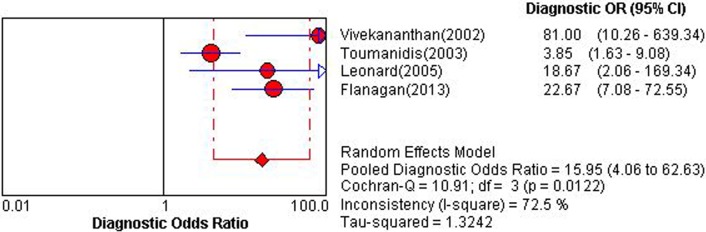
Pooled diagnostic odds ratio of index of myocardial performance for detecting acute cardiac allograft rejection. Effect sizes were pooled by random-effects models.

**Fig 7:**
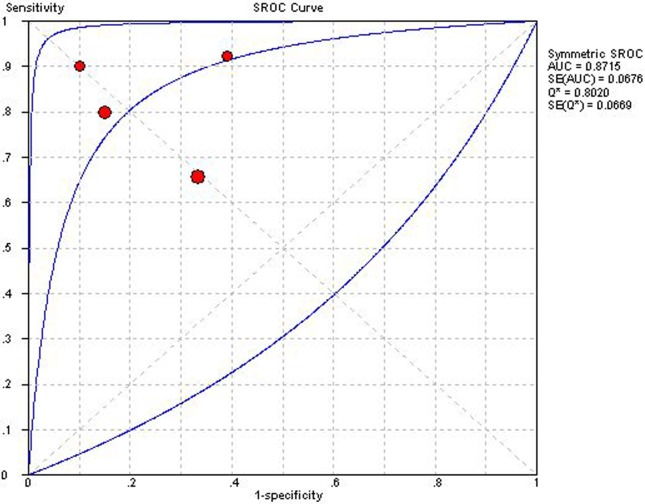
Summary receiver operator characteristic of index of myocardial performance. The figure shows a symmetric curve with an area under the curve of 0.8715 and standard error of 0.0676.

Four studies applying IMP could construct 2×2 table [21,24,41,42]. Overall diagnostic performance of IMP was shown in ***Figs. 6 and 7***. The Spearman correlation coefficient was computed as a result of 0.20 with a *P* value 0.80, which suggested the absence of a threshold effect. *I* ^2^ value of se, sp, PLR, NLR and DOR were 57.1%, 68.5%, 70.9%, 65.2%, and 72.5%, respectively, and corresponding *P* value of χ^2^ test were 0.07, 0.02, 0.01, 0.03, and 0.01, respectively. These results indicated a moderate degree of heterogeneity, and the source of heterogeneity was explored by sensitivity analysis. After removing the study of Toumanidis et al [41], which including detection for a relatively low grade of ACAR, IA and IB, homogeneity were achieved in se, NLR and DOR. However, sp and PLR still presented a moderate degree of heterogeneity. DerSimonian-Laird random effects model was used for pooling diagnostic indices. Pooled se, sp, PLR, NLP, DOR and AUC of SROC curve was 0.78 (0.70–0.86), 0.74 (0.66–0.81), 3.27 (1.76–6.06), 0.25 (0.12–0.54), 15.95 (4.06–62.63) and 0.871, respectively.

### Am

**Fig 8:**
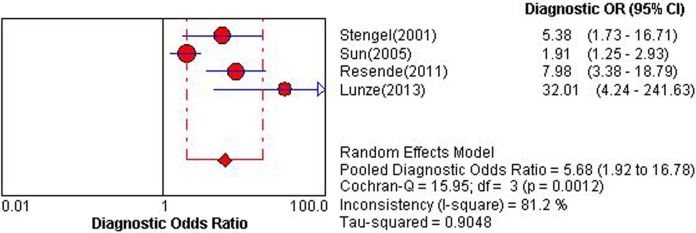
Pooled diagnostic odds ratio of late diastolic mitral annular motion velocity for detecting acute cardiac allograft rejection. Effect sizes were pooled by random-effects models.

**Fig 9:**
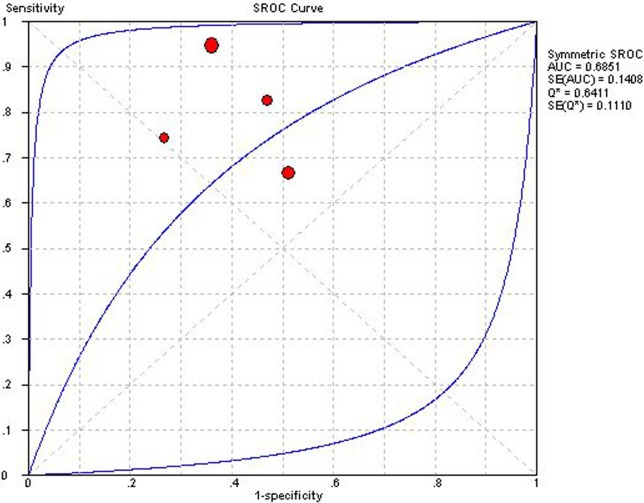
Summary receiver operator characteristic of late diastolic mitral annular motion velocity. The figure shows a symmetric curve with an area under the curve of 0.6851 and standard error of 0.1408.

Four studies applying Am could construct 2×2 table [25,36,37,38]. Overall diagnostic performance of Am was shown in ***Figs. 8 and 9***. The Spearman correlation coefficient was computed as a result of −0.40 with a *P* value 0.60, which suggested the absence of a threshold effect. For the existence of a high degree of heterogeneity, the source of heterogeneity was explored by using sensitivity analysis. After removing the study of Sun et al [38], which included detection of a relatively low grade of ACAR, IB, homogeneity was achieved in NLR and DOR. However, sp still presented a high degree of heterogeneity, and PLR and se showed a moderate heterogeneity. DerSimonian-Laird random effects model was applied for pooling diagnostic indices. Pooled se, sp, PLR, NLP, DOR and AUC of SROC curve was 0.72 (0.66–0.78), 0.60 (0.56–0.63), 2.01 (1.29–3.13), 0.38 (0.20–0.75), 5.68 (1.92–16.78) and 0.685, respectively.

### Major parameters summarization

A total of 46 articles (include 30 articles for meta-analysis) studied the correlation between a certain index and ACAR were enrolled for summarization [13–58]. The summary of correlation between 13 major parameters and ACAR was shown in ***Table 2***. Among eligible studies, 50% (9/18) articles showed a decrease in PHT or deceleration time (DT) was relevant to ACAR, and 45% (9/20) studies showed a decrease in IVRT was relevant to ACAR. An existence of correlation between IMP and ACAR was supported by 62.5% (5/8) studies, and correlation between Am and ACAR presented in 70% studies (7/10). Only 4 parameters, IMP, Am, Em and Sm, showed more than 50% of correlation with ACAR. Several parameters, such as ejection fraction, fractional shortening and pericardial effusion, which are universally acknowledged as poor diagnostic performance in ACAR, were not included in the summary. Moreover, several parameters like ventricular wall strain or stress were excluded, since only two or three studies assessed them.

**Table 2:** The summary of correlation between some major parameters and ACAR.

Study	PHT or DT	IVRT	IMP	A	E	E/A	Am	Em	E/Em	Em/Am	Sm	WT	LVMI
Angermann et al [13]												+	
Asante-Korang et al [14]							−	−		+			
Behera et al [15]	−	−		−	+			+	+				
Ciliberto et al [16]	+	+										+	
Desruennes et al [18]	+	+			−							−	
Dandel et al [19]	+	+						+			+		
Fauchier et al [20]	+	−											−
Flanagan et al [21]			+										
Kato et al [22]	−	−		−	−	−		+	−			−	
Leonard et al [24]	−		+									−	−
Lunze et al [25]							+	+	+		+		
Mouly-Bandini et al [26]	+	+											
Mankad et al [27]	−	+			−			+			+		
Moran et al [29]				−	−	−						+	+
Marciniak et al [30]	−	−		−	−			−			−	−	
Putzer et al [32]												+	+
Palka et al [33]								+	+				
Roshanali et al [35]	−	−			+		−	−				+	+
Resende et al [36]				+			+	+		−	+		−
Stengel et al [37]	−	−		−	−	−	+	−		−	−		−
Sun et al [38]	+	+		+	+	+	+	−	+		−	−	−
Sato et al [39]		−		−	−	−			−			−	
Sera et al [40]					−	−		−	−			−	−
Toumanidis et al [41]	+	−	+	−	−	−						−	−
Vivekananthan et al [42]			+										
Boyd et al [43]	−			−	−	−							
Burgess et al [44]		+	−										
Bader et al [45]			−	−	−		−	−	−			−	
Eun et al [46]		−			−		+	+	−	+			
Fabregas et al [47]							+	+					
Mannaerts et al [48]												−	
Miguel et al [49]	−	−	−			−		−	−			−	−
Neuberger et al [50]													−
Pauliks et al [51]							+	+			+		
Prakash et al [52]			+										
Rosenthal et al [53]					−							−	−
Störk et al [54]	+	+			+							−	
Spes et al [55]	−				−								
Stempfle et al [56]		−											
Valantine et al [57]	+	+			+								
Valantine et al [58]													
**Number of “+”**	9/18	9/20	5/8	2/11	5/19	1/9	7/10	10/18	4/10	2/4	5/8	5/18	3/13
**Percentage of “+”**	50%	45.0%	**62.5%**	18.2%	26.3%	11.1%	**70.0%**	**55.5%**	40.0%	50.0%	**62.5%**	27.8%	23.1%

A, late diastolic mitral inflow velocity; Am, Late diastolic mitral annular motion velocity; DT, deceleration time; E, early diastolic mitral inflow velocity; Em, early diastolic mitral annular motion velocity; IMP, index of myocardial performance; IVRT, isovolumic relaxation time; Sm, systolic mitral annular motion velocity; WT, ventricular wall thickness; +, the parameter correlates with rejection; −,the parameter does not correlate with rejection.

## Discussion

Due to sampling error of EMB associated with the inhomogeneous nature of ACAR, histological “false negative” ACAR is reported to occur in up to 20% of patients [59]. Furthermore, EMB is an invasive, expensive and uncomfortable procedure to patients. These drawbacks prevent more frequent monitoring and, thus, limit optimal immunosuppressive therapy in time. Despite many imaging modalities have been developed, the noninvasive detection of ACAR remains a clinical challenge.

Acute cardiac allograft rejection is characterized by a series of pathological alterations, including microvascular antigen deposition, myocyte edema, interstitial hemorrhage, inflammatory cellular infiltration, and varying degrees of myocardial necrosis. These alterations will impair myocyte function, especially diastolic function in the early stage [60]. As mentioned in our study, diastolic indices of echocardiography, including PHT, DT, IVRT, early diastolic mitral inflow velocity (E wave), late diastolic mitral inflow velocity (A wave), E/A, Em, Am, E/Em, Em/Am, were the most popular parameters for screening ACAR. When ACAR occurs, diastolic function is impaired as a result of ventricular wall stiffness through inflammatory cellular infiltration and myocardial edema, thus causing a reduction of left ventricular (LV) compliance and reflecting on these indices [18].

PHT is the time interval for the peak pressure gradient to reach its half level and is the same as the interval for the peak velocity to decline to a velocity equal to the peak velocity divided by $\sqrt{2}$ [61–63]. It is always proportionally related to DT. IVRT is an interval from the closure of the aortic valve to the onset of filling by opening the mitral valve [64]. The two indices can be used to systematically assess LV diastolic function. Decrease of PHT and IVRT in ACAR may reflect a decreased ventricular compliance due to the impairment of distensibility secondary to diffuse interstitial edema and inflammatory cellular infiltrate. Progression of edema and myocardial necrosis may increase the stiffness of elastic elements and shorten the rapid filling period [18]. A brisk increase LV diastolic pressure, which results in a rapid decrease of atrioventricular pressure gradient and premature closure of mitral valve, may lead to the decrease in PHT and IVRT [18]. However, the two diastolic indices, as is shown in our analysis, had a similarly poor performance for diagnosis of ACAR, only 40% of pooled sensitivity and 45% of correlation with rejection grade. Many factors, such as age, heart rate, loading condition, initial left atrial pressure, myocardial contractility and vasoactive agents, are known to affect LV filling [65–68,57]. Consequently, not only PHT and IVRT, but E wave and A wave are influenced secondary to the changes of LV filling. Second, a regional lesion in the early stage may only induce gentle alternation on diastolic indices. Moreover, for lack of autonomic regulation, donor heart will present with constant tachycardia and a restrictive ventricular filling pattern even in the absence of ACAR [37,69]. All these factors could explain the difficulties of these indices in the diagnosis of ACAR.

Am, Em and Sm derived from Doppler tissue motion parameters are new methods for evaluating left ventricular diastolic and systolic function. The myocardial contraction and relaxation velocities at the mitral annulus can more early and accurately detect ventricular dysfunction than PHT, IVRT, E and A wave [70,71]. During diastolic and systolic period, mean and peak motion velocity in different wall segments can be quantitatively acquired through tissue Doppler and pulse wave Doppler techniques. Analyzing the motion velocity of each ventricular wall during different cardiac cycle period might increase the diagnostic sensitivity, because this advantage, in theory, is in accord with the inhomogeneous pattern of rejection, and this regional index might be superior to the global indices like PHT, IVRT, E and A wave. In our study, 70% (7/10) researches showed the correlation between Am and ACAR. More than 50% articles supported Em and Sm were relevant to ACAR. However, the existence of high heterogeneity and rather poor pooled results indicate these parameters are not sufficient to distinguish rejections. The high degree of heterogeneity may come from clinical diversity (e.g. adults or children), methodological diversity (e.g. cutoff value or rejection grade), and statistical diversity [72]. We explored the source of heterogeneity and found Dendel et al, Resende et al and Lunze et al using a relative cutoff value achieved higher se (81.8%∼94.7%) and sp (64.4%∼94.1%) than Sun et al, Resende et al and Palka et al using an absolute cutoff value, with se (67%∼76.3%) and sp (49%∼73.8%). It might be more reasonable to choose relative alternation to baseline as cutoff value compared with absolute value.

IMP, another Doppler-derived index reflecting systolic and diastolic function, is defined as the summation of isovolumic contraction time and isovolumic relaxation time divided by ejection time [73]. This parameter partially fuses E and A wave, and is relatively not influenced by heart rate and ventricular geometry [74,75]. It also seems to be independent of alternations in preload, afterload and mitral regurgitation [76–78]. During an episode of ACAR, especially in the early stage and mild rejections, LV diastolic function may be compromised primarily, and systolic function is usually well preserved. With the development of the disease, systolic dysfunction occurs [57,79]. IMP combining the two indices has a promise to present ideal performance in monitoring rejection. In the meta-analysis, the diagnostic performance of IMP seems to be superior to the other indices, with a DOR of 15.95 and AUC of 0.871. The correlation between IMP and ACAR was supported in 62.5% studies (5/8). Miguel et al discovered IMP in HTX patients with and without rejection were significantly higher than that in healthy control group. In addition, IMP in HTX patients with rejection was higher than that in HTX patients without rejection, but not reaching statistical level, [49]. Burgess et al demonstrated an absence of significant change in IMP between rejection group and Non-rejection group. However, there was a significant increase in IVCT and a significant decrease in IVRT during rejection. It is possible that the decrease of IVRT is counterbalanced by IVCT prolongation, and this may lead to no significant change in IMP [44].

Wall thickness and left ventricular mass index subject to operator dependent errors, are relatively rough parameters in echocardiography [37]. Furthermore, an increase in wall thickness detectable by echocardiography due to myocardial edema is a rather late event [3]. Other indices like ventricular strain, strain rate and two dimensional integrated backscatter have shown more than 85% se and sp. However, the highly technical dependence and low reproducibility limit their application [60].

There were 7 studies testing two or more parameters and significantly improving the diagnostic value on ruling out therapeutically relevant ACAR with a higher sensitivity and negative predictive value (NPV) compared to single parameter test. In view of invasive and “false-negative” nature of EMB, multi-parameter echocardiography may have the potential of non-invasive tool for inclusion of all clinical suspected ACAR. Because parts of sub-clinical ACAR have the tendency to progress into severe rejection, and grade 1B have been combined into grade 2R in the revision of the international society for heart and lung transplantation [80], multi-parameter echocardiography might be considered as an alternative modality for surveillance sub-clinical ACAR. However, due to a small number of studies and methodological diversity, these combined parameters were not able to be evaluated comprehensively by meta-analysis.

### Limitations

Similar to other diagnostic meta-analysis, several limitations exist exactly in our study. First, studies ranged from 1988 to 2014, hence results may be affected by the progression of technique and device update. Second, only 4 studies applied 4 parameters in the meta-analysis. Because the number of eligible studies including other parameters is less than 3, hence, we cannot comprehensively evaluate their diagnostic performance. Third, the presence of high degree heterogeneity may have overestimated or underestimated the actually diagnostic accuracy. Moreover, 19 eligible studies did not mention double-blind principle, thus, it might increase the possibility of review bias; only 15 studies were confirmed to have enrolled patients consecutively that might cause selection bias; patients with atrial fibrillation or arrhythmia were excluded from some researches might also generate selection bias; all of eligible study published in English that could result in publication bias. Finally, the sample size of meta-analysis is relatively small. A larger sample size could acquire more reliable results.

## Conclusion

Although the existence of limitations, to our knowledge, this is the first meta-analysis to explore the diagnostic value of echocardiography in ACAR. The meta-analysis and systematic review demonstrate that no single parameter of echocardiography was able to provide a reliable performance in diagnosing ACAR. A result of echocardiography for ACAR should be comprehensively considered by physicians in the context of clinical presentations and imaging feature. Multi-parameter screening and several new techniques still need to be evaluated in the future.

## Supporting Information

S1 PRISMA Checklist (DOC)

S1 Fig Assessment of methodological quality according to QUADAS-2. (TIF)

S2 Fig Summary of methodological quality according to QUADAS-2. (TIF)
