# Supplementary figures and images for: Diagnostic Performance of Echocardiography for the Detection of Acute Cardiac Allograft Rejection: A Systematic Review and Meta-Analysis
Source: PLoS One. 2015 Mar 30;10(3):e0121228. doi: 10.1371/journal.pone.0121228 (PMC4378940; doi:10.1371/journal.pone.0121228)

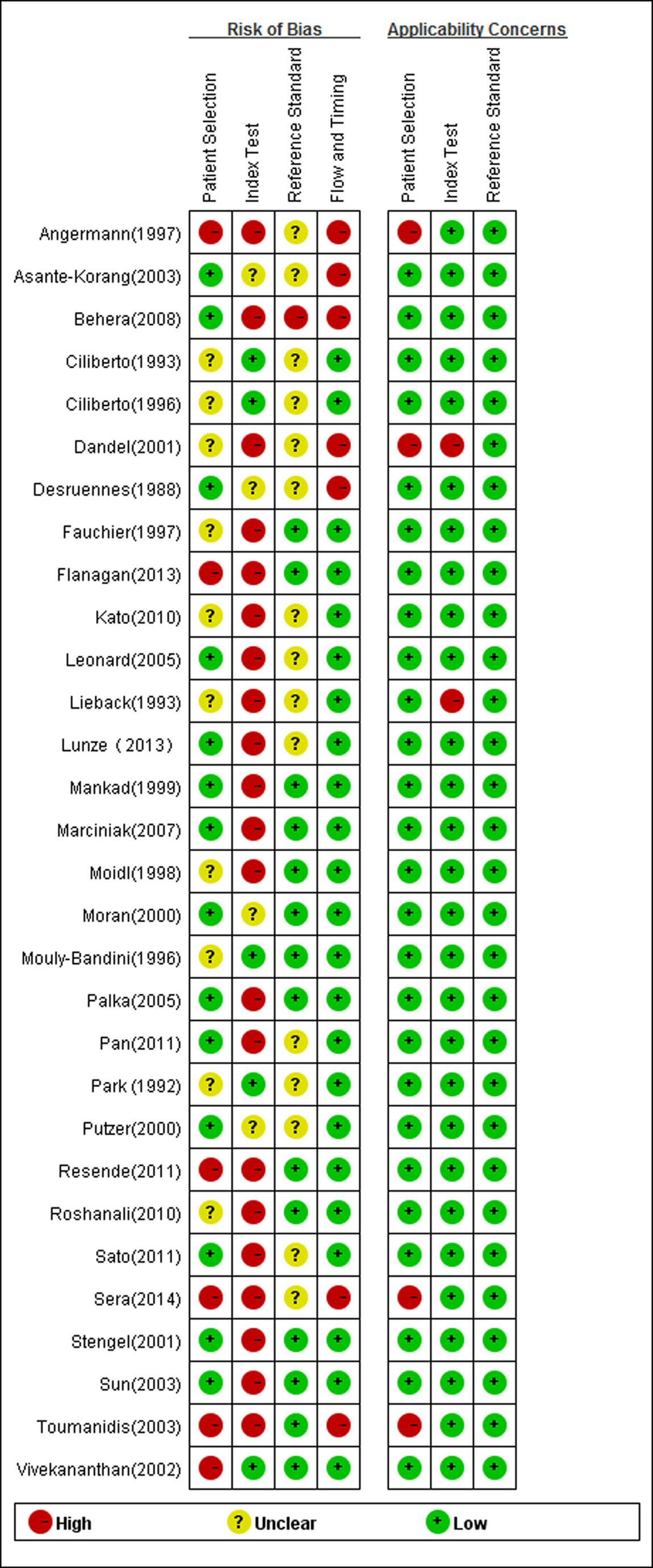

Supplement: S2 Fig — (TIF) [file pone.0121228.s003.tif]
